# Acceptance of the District Health Information System Version 2 Platform for Malaria Case-Based Surveillance By Health Care Workers in Botswana: Web-Based Survey

**DOI:** 10.2196/32722

**Published:** 2022-03-15

**Authors:** Kagiso Ndlovu, Kabelo Leonard Mauco, Mpho Keetile, Khutsafalo Kadimo, Refilwe Yvonne Senyatso, Davies Ntebela, Buthugwashe Valela, Clement Murambi

**Affiliations:** 1 Department of Computer Science Faculty of Science University of Botswana Gaborone Botswana; 2 Department of Health Information Management Faculty of Health and Education Botho University Gaborone Botswana; 3 Department of Population Studies Faculty of Social Sciences University of Botswana Gaborone Botswana; 4 Department of Library Services University of Botswana Gaborone Botswana; 5 National Malaria Programme Ministry of Health and Wellness Gaborone Botswana

**Keywords:** malaria case-based surveillance, district health information system, eHealth, technology acceptance model, Botswana, DHIS2, malaria, surveillance, public health, technology adoption, user acceptance

## Abstract

**Background:**

Similar to many low- and middle-income countries, Botswana has identified eHealth as a means of improving health care service provision and delivery. The National Malaria Programme (NMP) in Botswana has implemented the District Health Information System version 2 (DHIS2) to support timely malaria case reporting across its 27 health districts; however, the implementation of an eHealth system is never without challenges. Barriers to the implementation of eHealth innovations within health care settings may arise at the individual or organizational levels. As such, the evaluation of user perceptions of the technology is an important step that can inform its sustainable implementation. The DHIS2 was implemented without evaluating user perceptions beforehand; therefore, the Botswana Ministry of Health and Wellness was uncertain about the likelihood of acceptance and use of the platform.

**Objective:**

We aimed to determine the acceptance of the DHIS2 platform by the NMP in Botswana to gauge whether adoption would be successful.

**Methods:**

The study’s design was informed by constructs of the technology acceptance model. A survey, with items assessed using a 7-point Likert scale, and focus group discussions were undertaken with DHIS2 core users from 27 health districts and NMP personnel at the Ministry of Health and Wellness. The web-based survey was administered from August 3, 2020 to September 30, 2020.

**Results:**

Survey participants were core users (n=27). Focus group participants were NMP personnel (n=5). Overall, participants’ survey responses (frequently occurring scores of 7) showed their confidence in the DHIS2 platform for case-based surveillance of malaria; however, participants also noted some organizational issues that could compromise user acceptance of the DHIS2 platform.

**Conclusions:**

Participants’ responses indicated their acceptance of the DHIS2 platform; however, the consideration of factors related to organizational readiness could further enhance successful acceptance, and consequently, successful adoption of the platform by the malaria program in Botswana.

## Introduction

Botswana is among the countries that have made substantial progress, in the elimination continuum, in the fight against malaria. Between 2000 and 2015, the malaria incidence in Botswana fell dramatically, by 79%, from 0.136 in 2000 to 0.029 in 2015 [1]. Furthermore, mortality caused by malaria declined by 57%, from 1069 deaths in 2003 to 462 deaths in 2015 [2]. This reduction in malaria morbidity and mortality is mainly due to the government's implementation of malaria interventions [3,4] such as annual health care worker training on malaria case management to ensure accurate diagnosis and treatment of identified cases [5]. The transmission of malaria in Botswana is highly heterogeneous; transmission is mostly seasonal and unstable, occurring primarily between November and May during the rainy season [6] and primarily in the northern and eastern parts of the country [7]. However, malaria transmission in Botswana is very low compared to that in other sub-Saharan African countries [8]. Despite efforts toward malaria elimination, Botswana has not been successful in meeting its target of achieving zero indigenous cases [7]. Failure to meet the set target could be attributed to inadequate disease surveillance systems. This is because an adequate disease surveillance system will ensure that data collection, analysis, reporting, active case ﬁnding, and linkage to the response happens quickly to identify infections (symptomatic and asymptomatic), prevent ongoing transmission, and decrease the transmission efficiency of vectors. Similar to many low- and middle-income countries, Botswana has identified eHealth (ie, “the use of Information and Communications Technologies (ICT) for health” [9]) as a means of improving health care service provision and delivery, and other low- and middle-income countries have implemented eHealth systems for case-based surveillance of malaria [10,11]. Such trends continue to be spurred by the recent global awareness and application of eHealth technologies toward combating the current COVID-19 pandemic [12].

In Botswana, an eHealth system—the District Health Information System version 2 (DHIS2)—has been identified to improve malaria surveillance across the 27 health districts. The decision to consider DHIS2 was based on its documented benefits [13-15]. In spite of the availability of the DHIS2 platform for case-based surveillance of malaria, paper-based case notification forms continue to be utilized. Prior to the DHIS2, the National Malaria Programme (NMP) used a spreadsheet (Excel, version 2013; Microsoft Inc) for data capture, analysis, and reporting. At the district level, either monitoring and evaluation officers, a malaria focal person (the person who is responsible for management of the NMP in the district), or a community health nurse compiled information on malaria infections (patient name, age, date of diagnosis, physical address, the treatment offered, and method of detection) in the spreadsheet, based on presentations and diagnoses in the preceding week, which was then emailed to the NMP at the Ministry of Health and Wellness headquarters. In addition, a designated health care worker at each facility could complete a case notification paper form (in duplicate), which was sent to the district headquarters and to the NMP. The monitoring and evaluation officer compiled data from all 27 health districts in a spreadsheet arranged by year, month, district, and week.

Notwithstanding the potential benefits of the DHIS2 platform toward improving case-based surveillance of malaria in Botswana, the implementation of any eHealth system is never without challenges [16]. According to Ross et al [17], barriers to implementation of eHealth innovations within health care settings may arise at the individual, organization, or wider levels of the health care system. Acceptance by users (health care workers) has been documented as one of the key factors for successful implementation of an eHealth system [18]; therefore, evaluating user perceptions of the technology is an important step. The technology acceptance model features prominently among key theoretical approaches used to understand people’s intentions to accept various forms of information technology [19,20]. Many studies [20-25] that focus on explaining end user acceptance and predicting successful adoption of eHealth systems by health care organizations use the technology acceptance model—that an individual’s (1) acceptance of (intention to engage with) a technology depends on (2) perceived usefulness and (3) perceived ease of use [19-21]—as a basis. This model contends that a strong relationship exists between one’s intention to use technology and their actual usage behavior [19,20], and perceived usefulness is characterized by an individual’s belief that engaging a technology improves their job performance, while perceived ease of use refers to their belief that using technology requires minimal effort [19,20].

The DHIS2 platform for case-based surveillance of malaria in Botswana was implemented without evaluating user acceptance. Evaluation of user acceptance is necessary to inform sustainable implementation of technology. We aimed to evaluate user acceptance of the DHIS2 platform.

## Methods

### Study Design

Survey items and the focus group discussion guide were informed by constructs (acceptance of the technology, perceived usefulness, and perceived ease of use) of the technology acceptance model and were used to gain insights from users across the 27 health care districts in Botswana.

We developed a survey and focus group discussion guide, both in English. The drafts were first reviewed by a health care worker at the Ministry of Health and Wellness and by a medical librarian at the University of Botswana, after which, both tools were refined. The revised tools were then tested by 2 other health care workers, whose input resulted in further enhancement of the tools through improved branching logic. The final survey was administered using REDCap (Vanderbilt University [23]) forms from August 3, 2020 to September 30, 2020. The final version of the focus group questions was utilized to guide focus group discussions.

For both the survey and focus group, we used purposive sampling. The DHIS2 was put into place across the 27 health districts in Botswana, with each district having at least one core user (a health care worker whose main role involves dedicated interaction with the DHIS2 platform for the NMP). The sample consisted 1 core user from each health districts and NMP personnel at the Ministry of Health and Wellness headquarters who were part of a unit dedicated to the use of DHIS2 platform for the NMP in Botswana (the NMP coordinator, a health informatics officer, a software developer, and 2 monitoring and evaluation officers).

Participants from the 27 health districts were recruited by the NMP coordinator, and those who consented were sent a link to complete the survey. Only one author was responsible for accessing and managing the web-based database through the use of username and password. The survey consisted of questions about the type of facility where the respondent was based and the name of the district and of statements that assessed users' acceptance of the DHIS2 platform, perceived usefulness, and perceived ease of use, using a 7-point Likert scale. There were 30 close-ended statements and 4 open-ended questions.

Focus group discussions, 1 session which lasted for 1 hour, involved the 5 NMP personnel based at the Ministry of Health and Wellness, who were invited by email and text messaging to a videoconference (Google Meet, Google LLC; which was chosen because it was the only platform to which all participants had access). The purpose of the focus group discussion was explained to participants, after which, they were asked to provide consent to participate in the study. The focus group discussion was recorded (with participant permission) and later transcribed verbatim.

Descriptive statistics (mean, standard deviation, median, and mode) were calculated (Excel, version 2013, Microsoft Inc) for quantitative data (close-ended statement ratings). Qualitative data (from open-ended questions and focus group discussion) were analyzed using thematic analysis (NVivo, version 11; QSR International). Thematic analysis of participants’ responses was conducted to determine factors affecting the sustainable implementation of the DHIS2 platform for the case-based surveillance program. We used a deductive coding process [22]. A coding frame and a predefined list of descriptive codes were developed by 1 author and then discussed by all authors, which yielded 20 codes (“eHealth,” “mHealth,” “ICT,” “user acceptance,” “usefulness,” “ease of use,” “user interface,” “capacity development,” “internet connectivity,” “mobile devices,” “electronic medical record,” “electronic health record,” “security,” “privacy,” “confidentiality,” “interoperability,” “integration,” “data management,” “data analysis,” and “data reporting”). Transcriptions of the discussions were systematically and iteratively searched (in 2 cycles) for elements relevant to the 20 codes. Participants’ responses from focus group discussions, as well as open-ended responses from the survey, were matched to corresponding categories in the coding frame. This process was done iteratively to refine the alignment and identify high-level themes.

### Ethics

The study was approved by the Office of Research and Development of the University of Botswana (UBR/RES/IRB/BIO/224) and the Ministry of Health and Wellness (HPDME: 13/18/1). Participants provided informed consent. During data collection, we presented and explained the consent form to seek permission from potential participants. The consent forms also clearly explained the purpose of the study and provided assurance that data would be kept safe and deidentified. Participants were informed of their right to refuse to participate or withdraw from the study at any time.

## Results

The survey response rate was 89% (24/27). From a total of 27 DHIS2 core users (community health nurses: n=14; malaria focal person: n=9; health information and communication technology personnel: n=4), 24 responded (community health nurses n=13; malaria focal persons: n=7; health information and communication technology personnel: n=4). Of those who responded, 15 were from the district health management team, 3 were from public hospitals, and 6 were from public clinic facilities. Core users who did not respond were from public hospitals (n=2) and a public clinic (n=1). Participants most frequently responded that they agreed (4 items: mode 6) or strongly agreed (16 items: mode 7) with survey statements, which were aligned with technology acceptance model constructs (Table 1).

All 5 NMP personnel participated in the focus group and highlighted possible factors that could affect the sustainable implementation of the DHIS2 platform for case-based surveillance of malaria (Table 2). Ultimately, 5 themes were identified (governance, infrastructure, capacity building, data security, and usability).

**Table 1:** Acceptance of the District Health Information System version 2 based on the technology acceptance model.

Constructs^a^			Mean (SD)		Median		Mode
**Perceived ease of use**							
	Overall, I am satisfied with how easy it is to use DHIS2^b^	5.2 (1.9)		6		7	
	It was simple to use DHIS2	5.4 (2.0)		6		7	
	It is easy to find the information I needed	5.2 (1.7)		5		7	
	It was easy to learn to use DHIS2	5.3 (2.2)		7		7	
	I feel comfortable using DHIS2	5.7 (2.1)		7		7	
	The information (such as online help, on-screen messages, and other documentation) provided with DHIS2 is clear	5.1 (1.7)		5		7	
	The interface of DHIS2 is pleasant	5.7 (1.5)		6		7	
	DHIS2 gives error messages that clearly tell me how to fix problems	3.8 (2.2)		3		3	
	Whenever I make a mistake using DHIS2, I recover easily and quickly	4.6 (1.9)		4		3	
	The organization of information on the system screens is clear	5.8 (1.3)		6		6	
	The information provided for the system is easy to understand	5.3 (1.9)		6		7	
**Perceived usefulness**							
	I am able to complete my work quickly using DHIS2	5.1 (2.2)		6		7	
	I can effectively complete my work using DHIS2	5.5 (1.9)		7		7	
	I believe I became productive quickly using DHIS2	5.4 (1.9)		6		7	
	The information is effective in helping me complete the tasks and scenarios	5.4 (1.8)		6		7	
	DHIS2 has all the functions and capabilities I expect it to have	5.4 (1.5)		6		6	
	Overall, DHIS2 improved data management activities for the malaria program	5.6 (2.1)		7		7	
	Overall, DHIS2 improved data analysis of malaria cases	5.6 (1.6)		6		7	
	DHIS2 contributed to timely decision-making processes	5.9 (1.6)		6		7	
	Overall, DHIS2 improved timely reporting of malaria cases	5.8 (1.8)		6		7	
**Acceptance of technology**							
	I like using the interface of DHIS2	5.1 (1.9)		6		6	
	Overall, I am satisfied with the DHIS2 system	5.1 (1.9)		6		6	

^a^Responses were in the form of a Likert scale from 1 (strongly disagree) to 7 (strongly agree).

^b^DHIS2: District Health Information System version 2.

**Table 2:** Thematic presentation of factors affecting the sustainable implementation of the District Health Information System version 2 (DHIS2) platform for case-based surveillance of malaria.

Theme	Example quotes
Governance	“It adds on the already heavy workload, there should be people specific to do malaria data entry” “It brought about migration from manual system to electronic platform given that COVID-19 necessitates the use of technology.” “Have government employees being part of the system programming and maintenance/being part of the project or have ownership to the system” “Majority of health care workers should be shown the importance of using the health systems” “Cascade mentoring of DHIS utilization in facilities as facility based training” “Every time an update is introduced on the paper data collection tools (guidelines), the same should also be updated on the DHI2 system to make sure that the paper forms and the system are in sync at all times”.
Infrastructure	“Frequent network downtimes resulting in delays in reporting when the system is down” “It is not linked with other applications that the government has created to formulate reports and daily activities” “Data capture even when there is no connectivity and later sync when the internet connection is regained” “More gadgets should be availed for each facility and errors or SIM card malfunctions should be urgently addressed” “System linkage/interoperability with other existing systems” “Making sure internet is reliable at facilities”
Capacity building	“Requires more practice for better or improved competencies” “Capacity building to be strengthened for the local IT Officers” “Training should have frequent support visits” “Cascade mentorship programmes of DHIS utilization in facilities as facility-based training” “All health care workers should be trained and shown the importance of using the health information systems” “Topics covered to be taken step by step or explained in full with more practicals” “Training content coverage should be more than 80% practicals” “There should have been site visit immediately after training to assess situations on the ground”
Data security	“Use of authentication passwords and encryption algorithms for data security and privacy” “The link is not trustworthy”
Usability	“It picks everything you enter whether good or bad” “No free choice to select the period you want to view the data, all are fixed to daily, weekly, monthly, etc” “It's confusing sometimes as one can enter patients twice but not knowing” “Clients are attached to the diagnosing facility rather than the origin of infection” “If an error is made during entering of cases and you want to correct it, there isn't an option to edit especially on the date of diagnosis and notification” “Data management greatly improved for the intervention of positive malaria cases” “DHIS2 is easy to use as it simplifies data management and analysis” “It is pleasant, easier and quick to use, you can retrieve your information if you need to utilize it for something” “Timely data reporting” “Completeness of reports is achieved” “Easy analysis of data, using maps and graphs for visualisations” “Easy to formulate reports if used adequately and effectively” “It covered most aspects of malaria case-based surveillance” “DHIS2 has simplified malaria surveillance and indeed it is a very valuable tool” “Gives a good situational analysis of what is happening on the ground” “DHIS2 enables foursight mapping, by using the GPS coordinates to locate malaria case outbreaks or hotspots.” “Everything is now at our fingertips, we have data in the form of tables, charts and reports generated from the dashboard than prior to the DHIS2 where we used to manually manage the data using excel charts.” “We easily make decisions based on the data from the system”

## Discussion

### General

Overall, the study showed participants’ satisfaction with the DHIS2 platform, with the majority of responses indicating agreement (20/22; 4 items: mode 6; 16 items: mode 7) with positive statements based on technology acceptance model constructs (Table 1). Key themes—*governance*, *infrastructure*, *capacity building*, *data security*, and *usability*—related to an organization’s role in influencing technology acceptance were identified (Table 2).

The technology acceptance model constructs of perceived ease of use and perceived usefulness have been previously documented as positive influences toward technology adoption [24]. Moreover, *perceived ease of us*e has been defined as the extent to which a person believes that using a technology will be free of effort [24], and *perceived usefulness* is the belief that one’s utilization of information technologies will enhance one’s work performance [25]. For only 2 statements (addressing system usability) of the 22 survey items did the most common response by participants indicate that they somewhat disagreed (mode 3). Based on our findings, successful acceptance and adoption of the DHIS2 platform by the malaria program in Botswana could be assumed; however, NMP personnel highlighted some factors that could negatively influence acceptance of the DHIS2 for the malaria program. In an organization, factors that may affect sustainable implementation of the DHIS2 platform for case-based surveillance of malaria include the state of governance, the state of relevant infrastructure, the presence or absence of capacity building initiatives aimed at empowering potential users of the system, data security measures, and usability of the system (Table 2). As such, the application of project management practices by an implementing organization may be critical in ensuring sustainable implementation of the DHIS2 platform. Successful project management practices will ensure the fitness of a project for its political context (ie, in terms of organizational strategy, managership, and stakeholder management) [26]. Mlekus et al [27] highlighted that any organization planning for the successful acceptance and adoption of new technology should consider issues that fulfill user experience related to output quality, perspicuity, dependability, and novelty. Change management has been documented as one strategy that organizations could use to raise acceptance of new technology in a workplace and hence improve chances of successful adoption of the technology [28]. Change management is about supporting people through a process of change, and successful implementation of change is achieved when the systems, processes, tools, and technology of the change initiative are embedded in the new way in which health care providers do their work [29]. Ingebrigsten et al [30] identified 7 leadership behaviors that were associated with successful outcomes in Health Information Technology adoption: (1) communicating clearly about visions and goals, (2) providing support, (3) establishing a governance structure, (4) establishing training, (5) identifying and appointing champions, (6) addressing work process change, and (7) following up. Such leadership maybe necessary in addressing the issues highlighted in Table 2. Consequently, a lack of top management support and technology implementation strategy may play a negative role in influencing information and communication technology acceptance and adoption in any organization. Hu et al [31] noted that the ultimate success of an eHealth system in an adopting organization requires adequate attention to both technological and managerial issues. Therefore, for successful and sustainable implementation of an eHealth system, the health care organization should ensure availability of requisite resources and processes. In fact, failures of eHealth system implementation have been associated with a lack of eHealth readiness [32,33].

The technology acceptance model is one of the most widely used theoretical frameworks for predicting individuals’ likelihood to accept and adopt new technology [34]. The model is based on the assumption that when users perceive that a type of technology is useful and easy to use, they will be willing to use it [35]. However, some of our findings suggest the need for a model that considers the role of organizational readiness, the extent to which an institutional setting and culture support and promotes awareness, implementation, and use of eHealth [36], in influencing technology acceptance and hence the adoption of eHealth systems.

### Guidance

In line with key study findings, the following acceptance-related issues and associated mitigation strategies are proffered: Perception of usability can influence acceptance of an eHealth system. As such, the usability of the current DHIS2 platform for case-based surveillance of malaria should be enhanced as it could deter users from interacting with the platform. One focus group participant reported usability concerns about the DHIS2,

It's confusing sometimes as one can enter patients twice but not knowing.

Another participant reported that

If an error is made during entering of cases and you want to correct it, there isn't an option to edit especially on the date of diagnosis and notification.

Previous studies [37-40] have suggested enhancing system usability with user-friendly interfaces, real-time feedback mechanisms, and decision-support capabilities. It is, therefore, important to involve all key stakeholders, from design to implementation, by using a user-centered design approach [41]. User-centered design is also considered to be an important contributor to system usefulness and usability [41]. This is of importance since user-centered design creates a sense of ownership among end users who participated in the design and development process, thereby increasing their acceptance of the system

Digital health literacy is also a factor in technology acceptance [42]. Study participants highlighted the need for frequent training on the DHIS2 platform, with one stating

Training should have frequent support visits

while another suggested the DHIS2 platform

...requires more practice for better or improved competencies.

This could be achieved through continuous health human resource capacity-building programs as technology and user needs to evolve.

A potential data security consideration, the

...use of authentication passwords and encryption algorithms for data security and privacy...

was identified in this study, as well as, one security risk, that is,

The link is not trustworthy.

These issues require comprehensive well-documented regulatory approaches to facilitate protection of the highly sensitive clinical data. Given the limited documented data security and privacy best practices for open-source solutions, implementation of the DHIS2 platform in Botswana should be guided by and aligned to key national policy documents such as the Data Protection Act [43] or equivalent policy documents in other settings.

Focus group participants indicated that the DHIS2 could be enhanced by establishing

...system linkage/interoperability with other existing systems.

This followed a previous finding [44] highlighting that the DHIS2 is not linked with other applications that the government has created to formulate reports and daily activities. Interoperability of eHealth systems in Botswana and similar low- and middle-income countries should be strengthened through increased adaptation of universally available software, services, and content, such as the DHIS2 platform, as these are already endorsed by the World Health Organization to be interoperable [9]. Furthermore, the DHIS2 supports a web application programming interface that allows for integration with other databases and supports the development of an “Integrated Information Portal [45].” Lastly, it is important to view interoperability of eHealth systems as an ongoing process that can be improved over time.

### Conclusions

Health care environments in most low- and middle-income countries are generally data rich but information poor. In Botswana, the health care sector is compounded with vast amounts of clinical data which are seldom used for decision-making purposes. The DHIS2 is a platform that can improve data capture, analysis, and timely reporting, which will facilitate transitioning data to information. Evaluation of the DHIS2 platform for case-based surveillance of malaria by core users showed that it was accepted. There is, however, room to improve the usability of the current DHIS2 platform, through end user involvement and buy in, to ensure sustainable health human resource capacity building, to address security privacy and confidentiality issues, and to address interoperability, by considering guidance from the World Health Organization [9]. Our findings can be used to inform policy makers and health informatics leaders in Botswana and in similar low- and middle-income countries to successfully plan and implement effective eHealth platforms.

## Abbreviations

DHIS2: District Health Information System version 2
NMP: National Malaria Programme
